# The Influence of Bleeding of Cement Suspensions on Their Rheological Properties

**DOI:** 10.3390/ma13071609

**Published:** 2020-04-01

**Authors:** Tabea von Bronk, Michael Haist, Ludger Lohaus

**Affiliations:** Institute for Building Materials Science, Leibniz Universität Hannover, 30167 Hannover, Germany; t.von-bronk@baustoff.uni-hannover.de (T.v.B.); haist@baustoff.uni-hannover.de (M.H.)

**Keywords:** bleeding, cement suspension, rheology

## Abstract

Flowable concretes tend to segregate. The risk of segregation is particularly high when the concrete is vibrated during the compaction process. A well-known segregation phenomenon is the so-called “bleeding”. This is a rise of water to the surface of the freshly poured concrete due to the difference in density between the mixing water and the concrete’s denser solid components (aggregates, cement and additives). This type of segregation occurs particularly within the paste. The focus of this paper is, therefore, on the sedimentation behavior at the microscale of concrete and especially on the influence of this process on rheological properties of the cement paste. In addition to common bleeding tests of cement suspensions using standing cylinders, rheometric measurements were performed on the suspensions during the bleeding process. A measuring procedure was developed for the rheometric measurements of the sedimenting cement suspensions. The rheological properties of the investigated cement suspensions were determined at four specific measuring times and at four specific measuring heights (i.e., positions) each. With this method it could be shown that the cement suspensions are not homogeneous over their height and that bleeding has a great influence on the rheological properties of cement suspension.

## 1. Introduction

**Figure 1:**
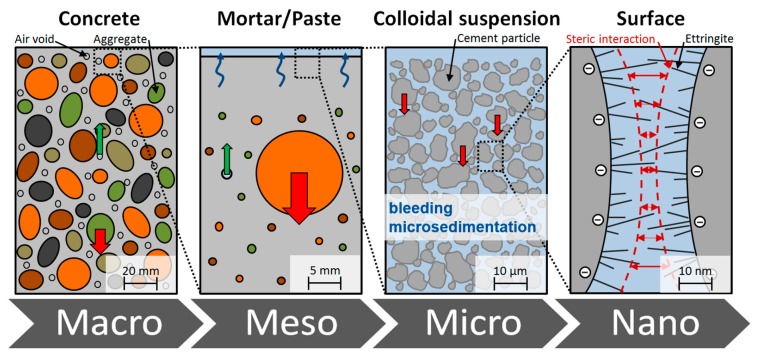
Illustration of concrete segregation phenomena on all scale levels of concrete, based on [2].

The sedimentation of solid particles such as aggregates, cement particles and supplementary cementitious materials (SCMs) due to their higher density compared to water and the development of a water film on the surface of fresh concrete is called segregation. This physical phenomenon is always present and occurs in varying degrees on all scale levels (see Figure 1): macro (concrete), meso (mortar) and micro (cementitious suspension). Segregation on the microlevel, i.e., in the fresh cement paste, is normally called ‘bleeding’ and describes a separation of water and cement particles. This separation process is caused by a sedimentation of cement particles in the suspension and is accompanied by a compaction of the particle structure [1].

The relevance of bleeding for the quality and durability of hardened concrete has been known since the early twentieth century, and the bleeding process has been the subject of numerous studies since then. Powers was one of the first, who studied the phenomenon of bleeding in detail. Powers [3] as well as Steinour [4] developed the first equations, based on Poiseuille’s Law of capillary flow, to determine the initial constant bleeding rate of cement pastes. In order to get a better understanding about the mechanism of settling in cement pastes, Wheeler and Chatterji calculated the density of a sedimenting (bleeding) fresh cement paste by X-ray radiography [5,6]. Using this method, they found that the bleeding process is accompanied by an increase in density of the consolidated cement paste and that a density gradient is formed over the height of the cement paste column. By using a gammadensiometer bench, Rosquoët et al. [7] detected an increasing density at the bottom of the cement paste column over time for high w/c-ratios (0.5–1.0), while the density at the top decreased.

Since cement pastes are suspensions with a high solids content, interparticle forces are inevitably involved in the bleeding process in addition to gravitational forces. Therefore, some authors stated that bleeding is rather a process of self-weight consolidation than of sedimentation [8,9,10,11]. Based on soil mechanics, the interparticle forces are considered as effective stresses in the paste [9]. In addition to the gravitational and interparticle forces, physical and chemical effects also play a role in the mechanism of bleeding in cementitious materials due to the hydration reaction. Josserand et al. [10] defined the bleeding of concrete as an ageing consolidation process in which the bleeding results mainly from the self-weight consolidation of the granular skeleton. For cement pastes and mortars, Tan et al. [8] presented a self-weight consolidation bleeding model that takes the hydration effect into account. Their results show that at a high w/c-ratio (by volume V_w_/V_c_ = 1.6; approximately w/c = 0.52), bleeding is due to a combination of sedimentation and consolidation and channels carrying water to the surface are often observed. In contrast, at a lower w/c-ratio (V_w_/V_c_ = 1.4), the authors concluded that bleeding is due to a combination of consolidation and little or no sedimentation occurs. Loh et al. [12] were able to determine that channelling is controlled by the particle concentration in the mixture and it occurs in the bleeding process of cement pastes and mortars whenever a mixture gets in a sedimenting regime and the structure of the particle network has not developed yet. Massoussi et al. [13] assume that the bleeding behavior of cement suspensions cannot be considered as resulting from consolidation of a soft porous media, but is of a heterogeneous nature and leads to the formation of preferential water extraction channels within the cement paste. Without preferential water extraction channels the bleeding would be negligible from their point of view. Moreover, for the process of bleeding they distinguished five distinct stages based on its kinetics: the induction period (low water extraction velocity), an accelerating regime (permeation increases due to the formation and percolation of water extraction channels), a constant water extraction rate period (constant water extraction velocity), a consolidation regime and finally a consolidated state (gravity is not able to further compact the constitutive cement grains).

The rheological behavior of fresh cement paste—and thus also of bleeding cement paste—plays a central role in controlling the rheology of fresh concrete [14,15]. A comprehensive insight into the rheology and physical interactions of cement suspensions is provided by Haist [16]. Essentially, cement suspensions are highly concentrated suspensions of solid particles of different sizes in which various interactions such as surface forces (or colloidal interactions), Brownian forces, hydrodynamic forces or various contact forces act between the particles, dispersed in a fluid phase [17]. The most important rheological parameters for describing the flow and deformation behavior of cement suspensions are the Bingham yield stress and the Bingham plastic viscosity. Whereas there are numerous investigations on the rheological properties of—more or less—homogeneous fresh cement pastes, hardly any information is available on the influence of bleeding on rheology.

The correlation between the yield stress and bleeding of cement suspension is very indirect because the yield stress results from a competition between Brownian motion and colloidal interactions whereas the competition between colloidal forces and gravity determines whether a cement suspension is stable or tends to bleed [18]. If the colloidal forces dominate, the suspension is stable, but if gravity dominates, sedimentation of particles occurs. Colloidal forces depend on the solid volume fraction (interparticle distance) and the average diameter of the grains (surface area). The study of Yang et al. [1] showed that the ionic strength of cement suspensions is always above a critical concentration for flocculation, both theoretically and experimentally, and therefore, they suggest that normal cement suspensions are always coagulated.

Basic requirements of rheological measurements are laminar flow within the sample under shear, steady state flow, wall adhesion and homogeneity of the sample [16]. Bhatty and Banfill [19] proved that the occurrence of sedimentation in cement suspensions during rheological measurements leads to large errors in the results and can only be limited by limiting the w/c-ratio or by adjusting the impeller geometry (angled blades). They also showed that sedimentation in cement suspensions can already occur with w/c-ratios as low as 0.28. With high w/c-ratios cement particles gravitationally may separate in the rheological experiments and also centrifugally [20]. An increase in the w/c-ratio results in additional water for the bleeding process [21]. The influence of w/c-ratio, admixtures and filler on sedimentation and bleeding of cement pastes was investigated by Peng and Jacobsen [22]: They proposed a conceptual model to study cement paste stability describing the development of bottom sediment, variable concentration, homogeneous zone, transition layer and bleeding. They also observed that the bleeding front is less sharp compared to classical bleeding observations and that it forms a soft transit layer below the transparent bleed water layer. Han and Wang [23] determined that bleeding of cement suspensions leads to an increased w/c ratio in the upper part of the cement paste, thus increasing the porosity of the hydrated cement paste in this area. In addition, bleeding and sedimentation lead to a different distribution of the hydration products (bottom: C-S-H gel and top: AFt-crystals), which is reflected not only in the mechanical properties but also in the gray scale value (color gradient) from dark to light along the bleeding direction.

The aim of the research presented here is to investigate and quantify the influence of sedimentation phenomena of cement suspensions on their rheological behavior. Thus, rheological measurements using a self-designed measuring geometry were carried out at four specific measuring depths and four specific measuring times in cement suspensions with a high bleeding potential due to their high water-to-cement-ratio (w/c-ratio ω).

## 2. Materials and Methods

### 2.1. Materials and Sample Preparation

In this study, cement pastes prepared with water to cement ratios ranging between 0.50 and 1.1 were investigated. The cement used in all investigations was provided by the Priority Program SPP2005 of Deutsche Forschungsgemeinschaft (DFG, German Research Foundation) [24]. It is an ordinary Portland cement (OPC, CEM I 42.5R; HeidelbergCement, Plant Ennigerloh) with a specific weight of 3.115 g/cm^3^, a mean particle size d_50_ = 14.8 µm and a Blaine value of 3600 cm^2^/g according to DIN EN 196-6 [25]. A comprehensive analysis of the cements properties is given in [26]. The cement paste was prepared by mixing the cement with demineralized water. Both constituents were stored at 20 °C prior to mixing. The masses of both components were dosed corresponding to the desired w/c-ratio. The cement suspensions were produced with an impeller mixer (Kenwood KM336 Chef Classic) according to the procedure given in Table 1. The volume of each sample was 1.5 L. After the sample preparation, a temperature measurement with a thermometer (Voltcraft^®^ DT-300) was immediately performed. The spread flow of the cement suspension carried out with the Haegermann cone according to DIN EN 1015-3 [27] was determined 9 min. after initial water–cement contact. For the subsequent rheological and microsedimentation characterizations, the pastes were homogenized at mixing intensity level 2 for one additional minute, starting 13 min. after the first water–cement contact.

### 2.2. Bleeding Measurements and Data Evaluation

**Figure 2:**
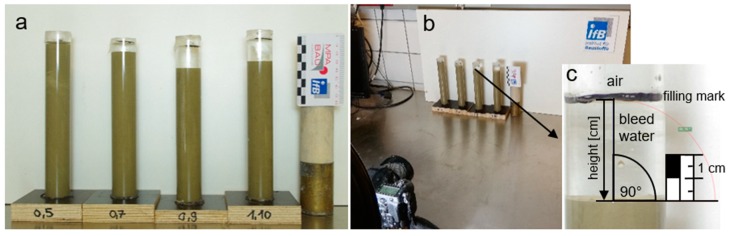
(**a**) Acrylic glass cylinders filled with cement suspension samples; (**b**) experimental set-up for the bleeding measurements on cement suspension samples and (**c**) example image of bleed water height.

After being homogenized, the cement suspension samples were poured into cylinders made of acrylic glass (see Figure 2a). The acrylic glass cylinders had a clear inner diameter of 26 mm and were filled to a height of 130 mm, which corresponds to a sample volume of 69 cm^3^. The cylinder opening was sealed immediately after filling with a sealing film (Parafilm^®^) to prevent evaporation. In order to record the development of the thickness of the water film on the surface of the sample, a camera was installed, which took a picture of the samples every 15 min. (see Figure 2b). Recordings of the samples were made over a period of not less than 180 min. for each sample. Image evaluation was carried out in order to quantify the height of the bleed water using the open source vector graphics program Inkscape (version 0.92.3).

### 2.3. Rheological Measurements and Data Evaluation

The rheological measurements on the cement suspensions were performed using a Thermo Scientific™ HAAKE™ MARS™ 60 Rheometer (Karlsruhe, Germany) equipped with a circulation thermostat, a cylindrical measuring cell for building materials and a specially designed measuring paddle. Details on the applied rheometer can be found in [16,28].

**Figure 3:**
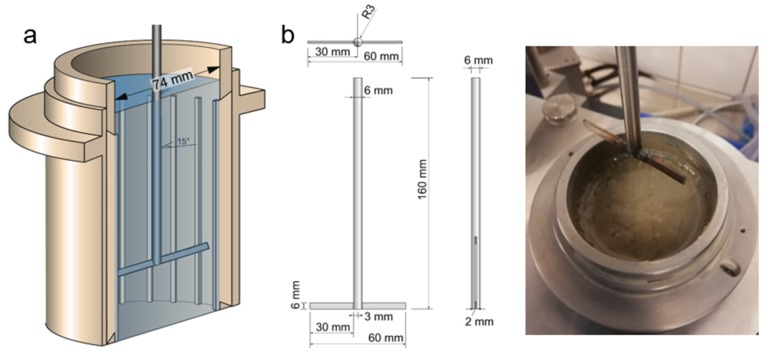
Geometry of measurement beaker (**a**) and of T-rotor (**b**) used for performing the rheological measurements.

The applied measurement cell consists of a cylindrical beaker with a clear diameter of 74 mm and a height of 150 mm. The walls of the beaker were serrated with a total of 24 stainless steel lamella, evenly distributed over the circumference of the beaker and protruding 1.5 mm into the beaker in order to prevent wall slippage. The geometry of the cell is detailed in Figure 3a. The beaker was filled with 548 cm^3^ of paste, corresponding to a filling height of 130 mm (compare bleeding test; filling height considers additional volume by protruding steel lamella). The required mass of cement suspension for the specified sample volume was determined by calculation from the sample’s density.

The rheological measurement was performed with a T-shaped rotor, consisting of a blade-like strip of stainless steel with a height of 6 mm and a thickness of 2 mm, connected to the bottom of a cylindrical axis, which is connected to the rheometer. This so-called T-rotor is shown in Figure 3b. The T-rotor is positioned relative to the filling height of the sample at any desired height by a precision step motor. Hereby, the bottom of the measurement cell designates the position level 0 mm. In this research, the position of the rotor given in mm designates the clear distance between the T-rotor and the bottom of the measurement cell.

In order to keep the sample temperature constant during the measurements and resting time, the circulation thermostat connected to the rheometer was set to 20 °C ensuring an outside cooling of the measurement beaker. Evaporation during measurement was prevented by a glass lid.

The rheological measurements were performed 15, 45, 90 and 180 min. after the initial water–cement contact and consisted of performing rotational measurements at four different positions, i.e., 110 mm (Position I), 80 mm (Position II), 40 mm (Position III) and 10 mm (Position IV), respectively, as measured from the bottom of the cell.

**Figure 4:**
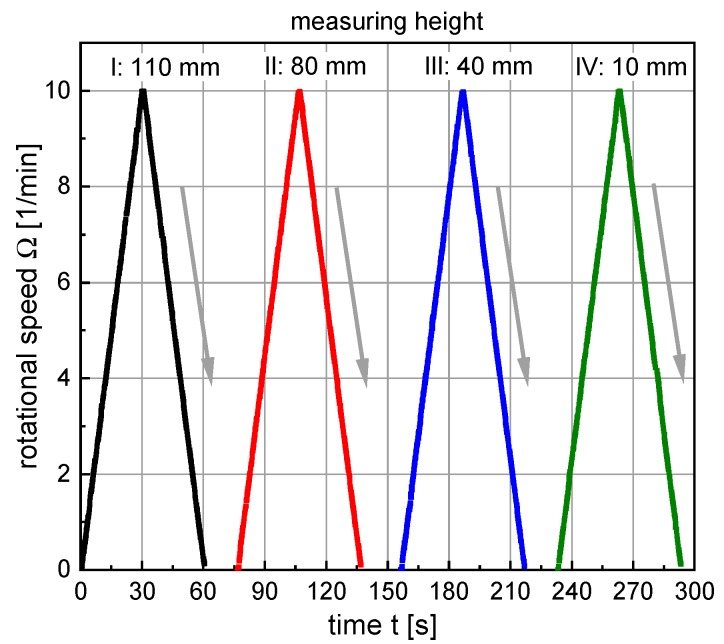
Rheometer measuring program. The downward ramp, marked by arrows, was used for data evaluation.

The measurement sequence consisted of (i) positioning the rotor at the desired position using the step motor and (ii) applying a rotational velocity-controlled ramp profile as shown in Figure 4, consisting of two linear ramp segments. The shear rate was increased linearly from 0 rpm to 10 rpm within 30 s and reduced from 10 rpm to 0 rpm within 30 s. The maximum shear rate was chosen as low as possible in order to keep the influence of the stirring movement of the T-rotor upon the sample condition to a minimum.

**Figure 5:**
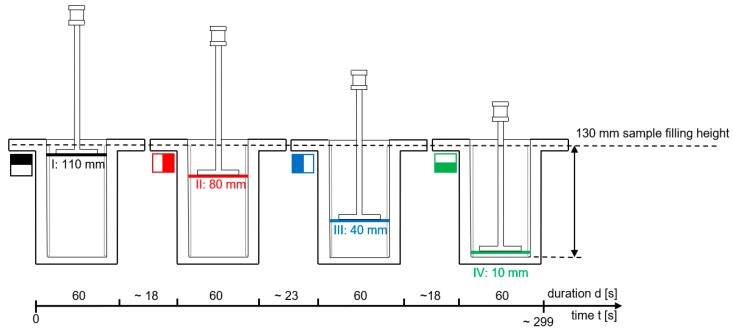
Illustration of the four measuring heights (I–IV) in a sample.

A graphical illustration of the four different measurements per sample age is shown in Figure 5 (note: color and symbol of the respective measurements). The immersion depth of the measuring paddle at the beginning of the rheometer measuring program was 110 mm. After completion of the first ramp element, the measuring paddle was vertically moved to a height of 80 mm. To avoid any disturbances of the suspension-structure the paddle was moved downwards with a very low velocity of approximately 2 mm/s. Correspondingly, the second ramp element started approximately 18 s after the end of the first segment, when the T-rotor had reached its new position of 80 mm. The procedure was then repeated at the third measuring height of 40 mm and finally at the fourth measuring height of 10 mm. The measuring sequence from top to bottom (110 mm–80 mm–40 mm–10 mm) was chosen so that any vertical transfer of particles by the measuring paddle occurred in the sedimentation direction and not in the opposite direction. Repeatability measurements clearly proved that due to the chosen geometry of the T-rotor (i.e., the very small width of the blade and the small diameter of the axis) such a vertical displacement of particles and thus an influence of the measurement on sedimentation could be neglected.

For data evaluation, the measured values resulting from the downward ramps of the rheometer measuring program were evaluated using a Bingham approach. Therefore, the flow curves in the range between 2 rpm and 9 rpm were evaluated by fitting Equation (1).

(1) $$T=T_{0}\;+\;\mu_{\mathrm{rel}.,\mathrm{pl}}\;\cdot \;\Omega$$

With: T = torque moment measured by the rheometer in Nm, $T_{0}=$ yield torque in Nm or µNm, $\mu_{\mathrm{rel}.,\mathrm{pl}}=$ slope of the Bingham curve, relative plastic viscosity in µNm·min, Ω = rotational speed in revolutions per min (rpm).

In order to evaluate the influence of wall friction on the T-rotor axis (note: the contact area between the rotor axis and the paste varies with different penetration depth of the rotor in the paste) onto the total torque T and thus onto the rheological values T_0_ and $\mu_{\mathrm{rel}.,\mathrm{pl}}$, reference measurements using a cylindrical rotor with identical shaft diameter but without the T-segment were carried out. Therefore, measurements at the four measuring times (15, 45, 90 and 180 min) and at the four measuring heights (positions I–IV) in a cement suspension with a w/c-ratio of 0.5 were carried out. This w/c-ratio was chosen as to cover the stiffest paste and thus the largest influence. For the investigated case, the shaft friction accounted for 1.3% of the total torque resistance and was therefore negligible.

## 3. Results

### 3.1. Time-Dependend Bleeding Behavior of Cement Suspensions

**Figure 6:**
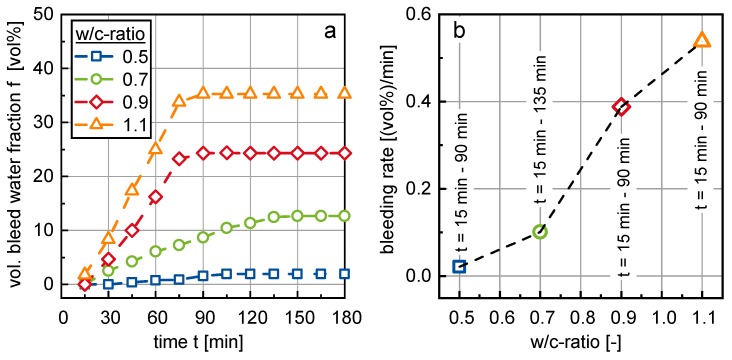
(**a**) Development of the bleed water volume over time and (**b**) bleeding rate as a function of the w/c-ratio.

Figure 6a shows the amount of bleed water in volume percent, which separated from cement suspension samples with w/c-ratios of 0.5, 0.7, 0.9 and 1.1, respectively, over a time period of 180 min. All samples exhibited more or less pronounced bleeding. The amount of bleed water in each sample was strongly dependent on the w/c-ratio. The higher the w/c ratio of the cement suspension, the more prone it was against bleeding.

All samples reached an equilibrium plateau of bleed water, depending on their w/c-ratio, at which the amount of bleed water did not increase further. In order to evaluate the velocity of the water separation process, the bleed curves from the start of the bleeding process (i.e., at t = 15 min) until the end of the bleeding process (i.e., when the curves reached the above-mentioned plateau) were fitted by a linear regression function. Figure 6b shows the slope of the bleed curves, in the following designated as the bleeding rate. The bleeding rate could be seen as approximately in linear dependence of the w/c-ratio.

Due to the bleeding process, the samples separate into a (compacted) cement suspension layer in the lower section of the measurement cylinder and a growing bleed water layer in the upper section of the cylinder. Both layers were clearly separated from each other (see Figure 2c). Due to this separation, the w/c-ratio $\omega_{insitu}$ and the phase content $\phi_{insitu}$ in the sedimented cement suspension layer no longer corresponded to the w/c-ratio $\omega_{0}$ and phase content $\phi_{0}$ of the initial cement suspension. The corresponding in situ values can be calculated according to Equations (2) and (3).

(2) $$\omega_{insitu}=\omega_{0}-f\cdot \left\{\frac{\omega_{0}}{\rho_{water}}+\frac{1}{\rho_{cement}}\right\}$$

(3) $$\phi_{insitu}=\frac{\phi_{0}}{1-f}$$

In Equations (2) and (3), f designates the volumetric bleed water fraction as shown in Figure 6a. $\rho_{water}=$ 1.0 g/cm^3^ and $\rho_{cement}$ = 3.3115 g/cm^3^ designate the densities of water and cement. The phase content ϕ can be calculated from the w/c-ratio ω according to $\phi =1/\left(\rho_{cement}\cdot \omega +1\right)$. 

**Figure 7:**
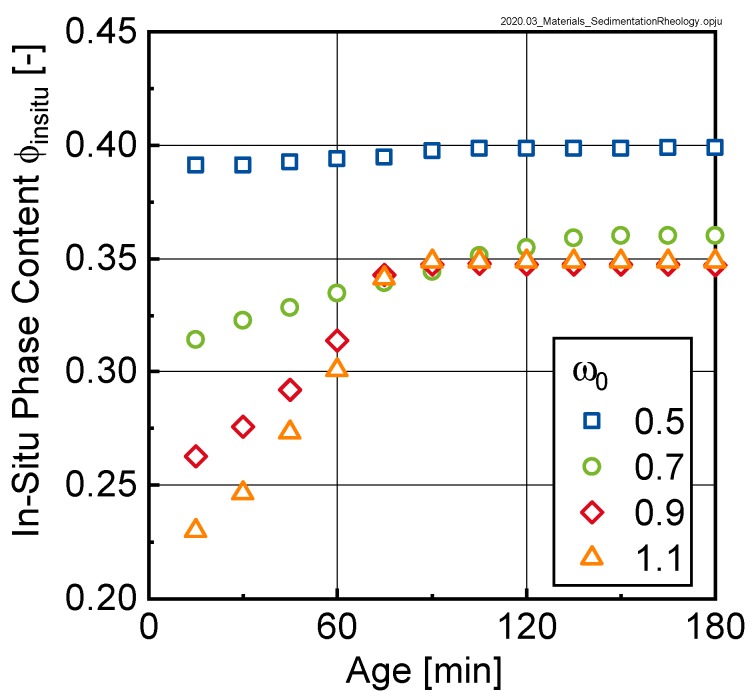
In-situ phase content $\phi_{insitu}$ of mixes with different initial w/c-ratios and corresponding initial phase contents $\omega_{0}$ as calculated according to Equation (3).

Figure 7 shows the temporal evolvement of the phase content for the investigated initial w/c-ratios $\omega_{0}$ of 0.5, 0.7, 0.9 and 1.1, respectively. As can be seen, the mixes can be classified into two groups. Whereas, the mix with $\omega_{0}$ = 0.5 shows only a very minor change in phase content, significant changes in the phase content over time can be observed for the mixes with higher initial $\omega_{0}$, i.e., for $\omega_{0}$ = 0.7, 0.9 and 1.1, respectively. The data further indicates that the packing density reached by sedimentation of particles was limited to values of approximately 0.35 for the mixes with $\omega_{0}$ ≥ 0.7. This result is in clear contrast to the in-situ packing density of the mix with $\omega_{0}$ = 0.5.

### 3.2. Rheological Behavior of Cement Suspensions during Microsedimentation/Bleeding

**Figure 8:**
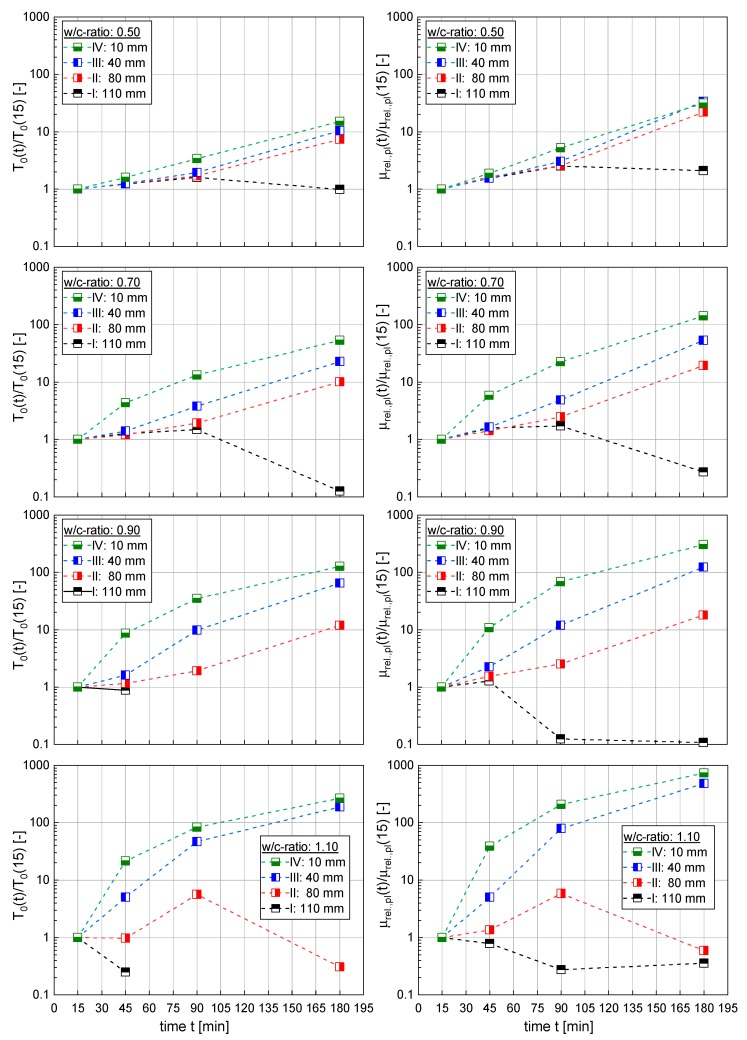
Temporal evolvement of the yield torque $T_{0}$ (left side) and the relative plastic viscosity $\mu_{\mathrm{rel}.,\mathrm{pl}}$ (right side) of cement suspensions (w/c-ratios: 0.5, 0.7, 0.9 and 1.1) at four measuring heights (I–IV) within the sample normalized by the initial values at a sample age of 15 min.

Figure 8 shows the temporal development of the yield torque $T_{0}$ and the relative plastic viscosity $\mu_{\mathrm{rel}.,\mathrm{pl}}$ of cement suspensions (w/c-ratios: 0.50, 0.70, 0.90 and 1.10) at four measuring heights (I–IV) within the sample. The rheological measurements on the cement suspensions were carried out as described in Section 2.3. The values for the yield torque $T_{0}$ and the relative plastic viscosity $\mu_{\mathrm{rel}.,\mathrm{pl}}$ for each position were normalized by the corresponding value at a sample age of 15 min (reference state). As can be seen, the rheological values increased continuously with time with some exceptions. These exceptions primarily occurred in strongly sedimenting cement suspensions with w/c-ratios of 0.90 and 1.10, respectively. Here, the thickness of the bleed water layer quickly reached values that were large enough, as for some measurements (especially for positions I and II) to occur in pure bleed water instead of cement paste.

The data in Figure 8 clearly shows that the rheological properties of the cement paste did not remain constant over time and were significantly influenced by the position within the sample. With increasing depth, i.e., the larger the distance from the top surface, the stronger the rheological properties increase, reaching values after 180 min between approximately 20 times up to approximately 200 times of the 15 min value, depending on the initial w/c-ratio. Comparing the change in yield torque T_0_(t)/T_0_(15) with the corresponding change in relative plastic viscosity µ_rel.,pl_(t)/µ_rel.,pl_(15) shows that sedimentation seems to influence both yield torque as well as relative plastic viscosity in a similar manner, yielding very similar changes for both characteristics.

Looking to the broader picture Figure 8 reveals that bleeding—and corresponding cement sedimentation—clearly affected the rheological behavior of the suspension. The rheological results however are not just the result of sedimentation only but are also affected by a chemical aging of the sample as well as it’s thixotropy, resulting from changes in the particle interaction behavior.

In order to separate the influence of the previously mentioned mechanisms, reference measurements on paste with an initial w/c-ratio of $\omega_{0}$ = 0.5 were carried out. The rheological properties of this paste were measured according to the procedure described in Section 2.3, however for this series, the paste was stirred in a mixer prior to each measurement, thus avoiding sedimentation and restructuring of the paste. Using these results, the sedimentation results shown in Figure 8 can be corrected for the chemical ageing of the sample.

**Figure 9:**
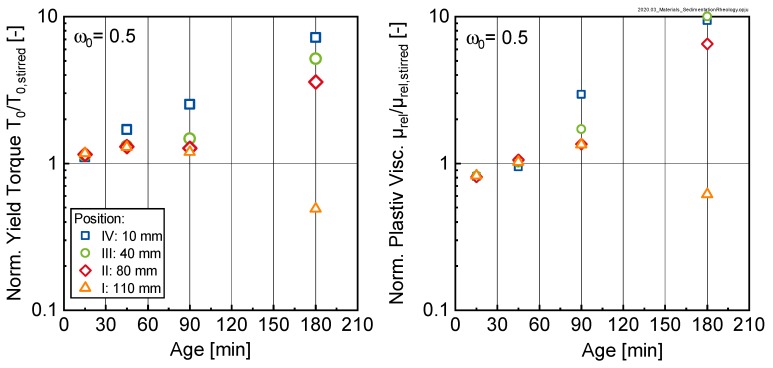
Yield torque (left) and relative plastic viscosity (right) of bleeding cement paste samples with an initial w/c-ratio $\omega_{0}$ = 0.5 normalized by the corresponding values of identical pastes, which, however, were remixed (index ‘stirred’) prior to the measurement.

As can be seen in Figure 9, for the considered sample with $\omega_{0}$ = 0.5, the normalized yield torque and the normalized relative plastic viscosity in the sedimented layer (position IV: 10 mm) increased exponentially with time. However not all positions in the paste seemed to be affected in a similar manner. For this cement paste ($\omega_{0}$ = 0.5) the lowest layer (i.e., position IV) seemed to compact continuously whereas for the adjacent layers III, II and I the compaction process stagnates after some initial set before becoming more pronounced in these layers after 180 min. 

## 4. Discussion

**Figure 10:**
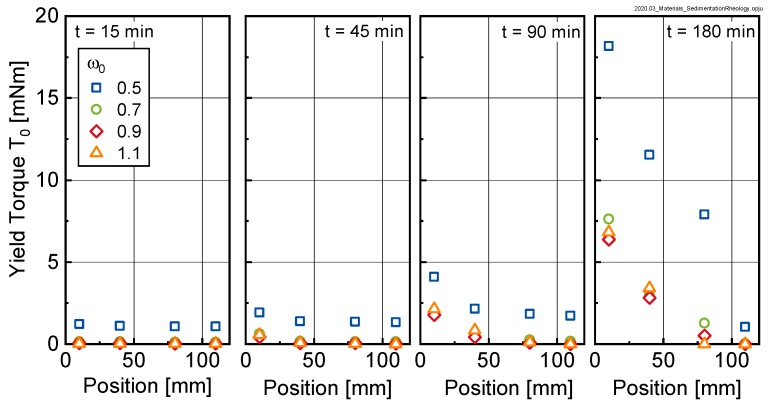
Yield Torque T_0_ measured at different positions for different points in time for initial w/c-ratios $\omega_{0}$ of 0.5, 0.7, 0.9 and 1.1, respectively.

**Figure 11:**
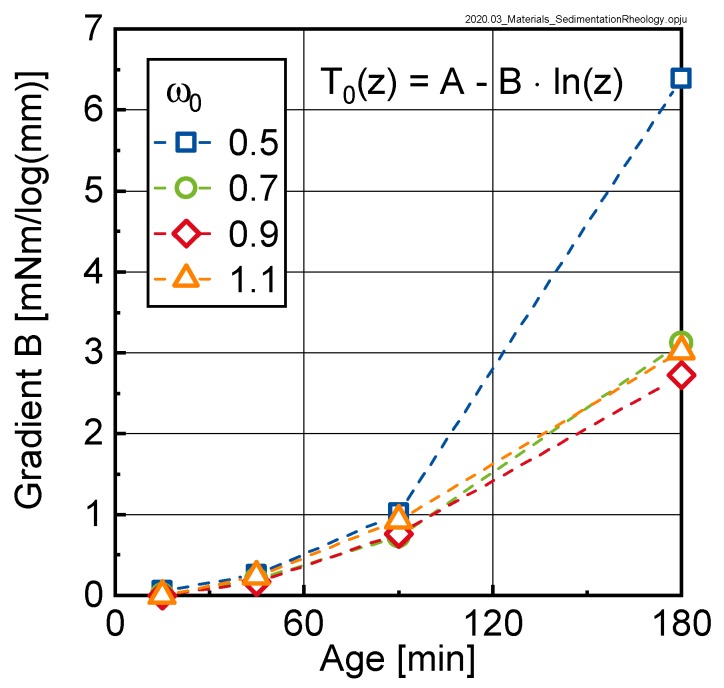
Gradient B according to Equation (4) of the yield torque T_0_ vs. height z curves (see Figure 10) as a function of sample age for the investigated pastes (initial w/c-ratios $\omega_{0}=$ 0.5, 0.7, 0.9 and 1.1).

The amount of bleed water is an indicator for the bleeding tendency of the sample. Bleeding however is only the macroscopic sign of a much more complex mechanism, in which microscale cement particles sediment due to gravity. A key question to be answered in this paper was how this sedimentation process affects the rheological properties of the cement suspension. The results shown in Section 3 clearly indicate that the sediment, i.e., the mixture of sedimented particles and water, was not homogeneous, which is in accordance with the literature. The rheological properties of this resulting suspension varied over the height of the sample. Figure 10 shows that bleeding, i.e., the separation of water on the top of the sample, led to an overall shift in the yield torque to higher values with increasing age. Fitting the data in Figure 11 using a logarithmic approach (see Equation (4)) yielded the results shown in Table 2.

(4) $$T_{0}\left(z\right)=A-B\cdot \ln\left(z\right)$$

In Equation (4), T_0_ designates the yield torque of the paste at a certain point in time and *z* stands for the position in the sample in mm measured from the bottom of the beaker. A and B are regression parameters.

The parameter B in Equation (4) designates the gradient of the rheological properties over the height of the sample and is displayed as a function of time for the investigated pastes in Figure 11.

As can be seen, the gradient of the yield torque over the height of the sample significantly increased with time, documenting the sedimentation process. A similar trend was observable for the relative plastic viscosity (not shown here). From the approximation with Equation (4), it was concluded that the rheological properties of the suspension varied over the height of the sample in a logarithmic manner. The data further shows that the gradient in rheological properties was quasi-independent of the initial w/c-ratio $\omega_{0}$, except for sedimentation after 180 min. Here we clearly saw that the gradient in rheological properties for $\omega_{0}$ = 0.5 was much more pronounced than for the samples with higher initial w/c-ratio.

**Figure 12:**
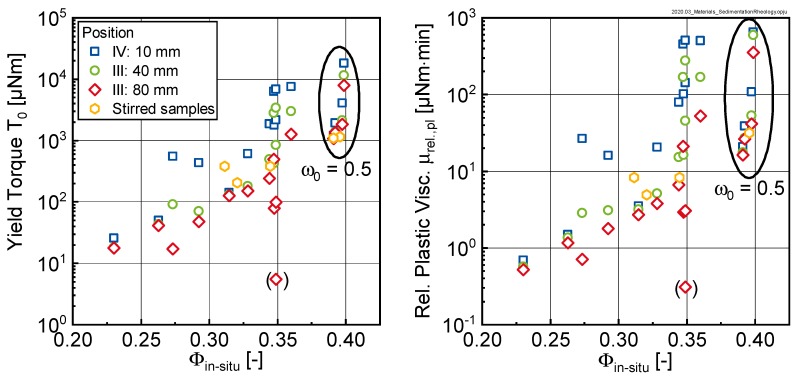
Yield torque T_0_ (left) and relative plastic viscosity µ_rel.,pl_ (right) as a function of the in-situ phase content $\phi_{insitu}$ for different measurement positions I–IV.

The reasons for the comparable behavior of the curves with initial w/c-ratios $\omega_{0}$ ≥ 0.7 could be found in Figure 12. Here, the yield torque and relative plastic viscosity were plotted as a function of the in-situ phase content $\phi_{insitu}$ as calculated using Equation (3). Clearly two groups could be distinguished: pastes with initial $\omega_{0}$-values of 0.7, 0.9 and 1.1 (corresponding to $\phi_{insitu}$-values of between 0.20 and 0.35) and a second group with $\omega_{0}$ = 0.5 (corresponding to $\phi_{insitu}$-values of approximately 0.4).

For the first group, the rheological properties increased with increasing phase content up to a limit phase content of approximately 0.35 (compare Figure 7). When this limit phase content was reached, a pronounced increase in the rheological properties (note logarithmic scaling) could be observed, which however was not reflected in changes of the in-situ phase content. This was explained by the fact that $\phi_{insitu}$ as calculated according to Equation (3) only accounted for the segregated water (i.e., the bleed water) but did not consider the gradient in the sediment itself. Interestingly, samples with $\omega_{0}$ ≥ 0.7 even in the sedimented state did not seem to reach the same packing density as the sample with $\omega_{0}$ = 0.5. For this second group, also here the rheological properties seemed to be influenced by the phase content.

## 5. Conclusions

The goal of the study described in this paper was to evaluate the effect of bleeding and corresponding microsedimentation onto the rheological properties of fresh cement paste, expressed by the yield torque (corresponding to the Bingham yield stress) and the relative plastic viscosity (corresponding to the Bingham plastic viscosity). Therefore, sedimentation tests were carried out on fresh cement pastes with different initial w/c-ratios at constant ambient conditions over a time frame of 180 min. The change in rheological properties over time and over the height of the sample was measured.

The results clearly show that the sedimentation process led to a pronounced increase in the rheological properties. This increase depended on the position in the sample, with the rheological parameters being in a logarithmic relationship with regard to the vertical position. The slope of this logarithmic line increased with time, however, it is independent of the initial w/c-ratio. The results further indicate that the packing density generated by bleeding seemed to be limited to a maximum threshold value of 0.35. Further investigations have to show whether the change in rheological properties over the height of the sample are a function of a further change in packing density over the height of the sample in the sedimented state or whether the observed changes are primarily caused by an increased physical interaction of the sedimented particles.

With regard to rheological measurements on cement paste in general, the results shown in this study clearly proved that such rheological measurements were strongly influenced by sedimentation and bleeding processes. For very liquid samples thus the measurement duration should be kept as short as possible. The data shown in Figure 11 allowed us to quantify this time period. When looking to the practical application of concrete it must be kept in mind that bleeding will be enhanced by the compaction of the concrete. This question will be studied in future research.

## Figures and Tables

**Table 1:** Mixing procedure for the cement suspensions.

Time (s)	Intensity (Level)	Process Step
60	1	pre-mixing of the dry cement
15	1	addition of water ^1^
45	1	mixing
90	-	mixing pause and removing of caking in the mixing bowl
60	2	mixing
30	-	mixing pause and removing of caking in the mixing bowl
120	2	mixing

^1^ Start of time measurement.

**Table 2:** Regression parameters A and B as determined by fitting Equation (4) to the data shown in Figure 11.

Age	ω	A	Error A	B	Error B	Adj. R^2^
15	0.5	1 335	26.1	58	6.7	0.961
	0.7	161	1.5	8	0.4	0.993
	0.9	60	1.1	4	0.3	0.986
	1.1	34	2. 8	4	0.7	0.909
45	0.5	2 454	244.4	255	63.0	0.837
	0.7	1 028	201.3	200	51.9	0.822
	0.9	791	161.7	171	41.7	0.841
	1.1	1 054	173.7	236	44.8	0.899
90	0.5	6 281	697.4	1 018	179.8	0.912
	0.7	3 438	489.1	727	126.1	0.915
	0.9	3 428	385.7	759	99.4	0.950
	1.1	4 285	208.5	932	53.6	0.990
180	0.5	33 738	5 638.6	6 387	1 453.4	0.859
	0.7	14 730	478.3	3 120	123.3	0.995
	0.9	12 673	504.3	2 721	130.0	0.993
	1.1	13 967	1 460.1	3 026	376.4	0.955
